# Total fecal IgA levels increase and natural IgM antibodies decrease after gastric bypass surgery

**DOI:** 10.1111/apm.13268

**Published:** 2022-08-26

**Authors:** Natalie Istomin, Mari‐Anne Härma, Ramin Akhi, Antti E. Nissinen, Markku J. Savolainen, Krishna Adeshara, Markku Lehto, Per‐Henrik Groop, Vesa Koivukangas, Janne Hukkanen, Sohvi Hörkkö

**Affiliations:** ^1^ Medical Microbiology and Immunology, Research Unit of Biomedicine University of Oulu Oulu Finland; ^2^ Medical Research Center Oulu Oulu University Hospital and University of Oulu Oulu Finland; ^3^ Nordlab Oulu University Hospital Oulu Finland; ^4^ Folkhälsan Institute of Genetics, Folkhälsan Research Center Helsinki Finland; ^5^ Abdominal Center, Nephrology University of Helsinki and Helsinki University Hospital Helsinki Finland; ^6^ Clinical and Molecular Metabolism, Faculty of Medicine Research Programs University of Helsinki Helsinki Finland; ^7^ Research Unit of Internal Medicine and Biocenter Oulu University of Oulu Oulu Finland; ^8^ Department of Surgery Oulu University Hospital Oulu Finland

**Keywords:** Roux‐en‐Y gastric bypass surgery, type 2 diabetes, natural antibodies, immunoglobulins, obesity

## Abstract

Obesity is associated with low‐grade inflammation and increased systemic oxidative stress. Roux‐en‐Y gastric bypass (RYGB) surgery is known to ameliorate the obesity‐induced metabolic dysfunctions. We aimed to study the levels of natural antibodies in feces, before and 6 months after RYGB surgery in obese individuals with and without type 2 diabetes (T2D). Sixteen individuals with T2D and 14 non‐diabetic (ND) individuals were operated. Total IgA, IgG and IgM antibody levels and specific antibodies to oxidized low‐density lipoprotein (oxLDL), malondialdehyde‐acetaldehyde adducts (MAA adducts), *Porphyromonas gingivalis* gingipain A hemagglutinin domain (Rgp44) and phosphocholine (PCho) were measured using chemiluminescence immunoassay. Total fecal IgA was elevated, while total IgM and IgG were not affected by the surgery. Fecal natural IgM specific to oxLDL decreased significantly in both T2D and ND individuals, while fecal IgM to Rgp44 and PCho decreased significantly in T2D individuals. A decrease in IgG to MAA‐LDL, Rgp44 and PCho was detected. RYGB surgery increases the levels of total fecal IgA and decreases fecal natural IgG and IgM antibodies specific to oxLDL. Natural antibodies and IgA are important in maintaining the normal gut homeostasis and first‐line defense against microbes, and their production is markedly altered with RYGB surgery.

## INTRODUCTION

Obesity and diabetes are increasingly important health concerns worldwide. These diseases associate with atherosclerotic cardiovascular diseases, dyslipidemia, chronic low‐grade inflammation, and increased oxidative stress. Obesity has also been shown to associate with increased premature aging and early mortality [1].

Natural antibodies are present prior to birth and considered to possess an important function as first line of immune defense of the newborns. These are mainly IgM isotype, and also IgG and IgA isotypes have been described [2]. Natural antibodies are germline‐encoded and have a broad reactivity against well‐conserved epitopes, and they recognize ligands of varying biological composition in microbes and altered‐self material. A well‐characterized group of antigenic epitopes for natural antibodies is oxidized lipids in oxidized low‐density lipoprotein (oxLDL) [3]. Oxidation of LDL phospholipids creates aldehydes, such as malondialdehyde (MDA), which is relatively unstable and further reacts with acetaldehyde to form malondialdehyde‐acetaldehyde (MAA). These MAA adducts have been shown to be targets for natural antibodies, and studies have revealed cross‐reactive antigenic properties between oxLDL and certain microbial virulence factors such as *Porphyromonas gingivalis* gingipain A hemagglutinin domain (Rgp44), bacterial cell wall component phosphocholine (PCho) and *Aggregatibacter actinomycetemcomitans* heat shock protein 60 (AaHSP60) [4, 5, 6, 7]. We have reported natural antibodies binding to oxLDL and microbial cross‐reactive virulence factors in blood, cerebrospinal fluid and saliva, and that they may associate positively with the disease burden [3, 8, 9, 10, 11].

Roux‐en‐Y gastric bypass (RYGB) surgery was developed over 50 years ago to aid weight loss and reduce comorbidities such as diabetes in people with obesity [12]. A meta‐analysis comparing non‐surgical treatment of obesity to gastric bypass surgery documented greater body weight loss and higher remission rate of diabetes with surgery [13]. Several studies have documented that gastric bypass surgery reduces levels of inflammatory and oxidative stress markers [14, 15, 16, 17] and that elevated levels of oxLDL have been reported in diabetes and metabolic syndrome [18, 19]. Levels of oxLDL decrease after bariatric surgery [20, 21] but there are no prior studies on the effect of bariatric surgery on natural antibodies or total immunoglobulin levels.

The aim of the present study was to investigate how RYGB surgery affects the levels of natural antibodies in the gut, in people with obesity. Additionally, we evaluated whether diabetes as comorbidity played significant role in modifying the gut natural antibody levels.

## MATERIAL AND METHODS

### Study subjects

Thirty obese individuals were recruited for the study during the years 2010–2014 in the Oulu University Hospital, Finland. The inclusion criteria were a medical indication for RYGB and an age between 18 and 65 years. Feces and plasma samples were collected before and approximately 6 months after the surgery from two study groups: [1] obese type 2 diabetic (T2D) individuals without insulin treatment (N = 16) and [2] obese non‐diabetic (ND) individuals (N = 14). For comparison, six healthy, non‐diabetic, individuals with BMI <30 kg/m^2^ were recruited from subjects undergoing routine gastroscopy for a non‐chronic benign disease. Additional excluding criteria for all study participants were oral cortisol medication, insulin treatment, long‐lasting antibiotic medication, a chronic inflammatory disease (e.g., inflammatory bowel syndrome or rheumatoid arthritis), celiac disease and malignancy. More detailed description of study protocol has been published earlier [22].

The study followed the ethical standards of Helsinki Declaration and was approved by the Ethics Committee of the Northern Ostrobothnia Hospital District. A written informed consent was obtained from each study participant. The trial was registered at ClinicalTrials.gov as NCT01330251.

### Fecal sample collection and preparation for immunoassays

Study subjects were instructed to collect the fecal samples at home, divide them into four aliquots, refrigerate (+4 °C – +8 °C) immediately and deliver samples to the Research Unit within the same day in a provided polystyrene foam container together with frozen gel packs. If not possible to deliver the samples within same day, the subjects were instructed to home‐freeze the samples (−18 °C to −20°C) and deliver them within 2 days. The samples were then stored at −70 °C for further analysis.

The fecal samples were prepared for immunoglobulin measurements as described earlier, with minor modifications [23]. Approximately 500 mg of feces was weighed and homogenized into ice cold elution buffer (PBS‐buffer with 0.05% NaN3 and 0.27 mM EDTA) to obtain 0.1 mg/mL concentration. The fecal samples were then allowed to solubilize for one hour at +4°C assisted with two to four short vortexing periods. The samples were centrifuged for 20 min at 1500 *g* (+4°C), and the supernatants were transferred into tubes containing phenylmethylsulphonyl fluoride and Sigma‐FAST protease inhibitor cocktail (S‐8830; Sigma‐Aldrich, St. Louis, MO, USA) as additional protease inhibitors. After re‐centrifugation for 10 min (+4 °C), the final supernatants were stored at −20°C.

### Chemiluminescence immunoassays and specific antigens

Total fecal IgA, IgG and IgM immunoglobulin levels were measured using chemiluminescence immunoassay as described [24, 25, 26]. Specific antibody binding to five different oxLDL and bacterial cross‐reactive virulence factors (copper‐oxidized low‐density lipoprotein [CuOx‐LDL], MAA‐LDL, malondialdehyde‐acetaldehyde bovine serum albumin [MAA‐BSA], PCho and Rgp44) were measured with chemiluminescence immunoassay. The antigens and their sources are listed in detail in Table 1.

**Table 1:** Oxidized‐LDL and cross‐reactive bacterial virulence factor antigens tested for binding by specific antibodies of fecal samples

Short name	Description of the antigen	Source	Key references
CuOx‐LDL	Low‐density lipoprotein oxidized *in vivo* by exposure to copper ions. Contains numerous different kinds of antigenic epitopes. ApoB100 apolipoprotein is usually markedly fragmented during the oxidation process	In‐house	[6, 43, 56]
MAA‐LDL	Low‐density lipoprotein modified with malondialdehyde‐acetaldehyde. Contains intact LDL particles with apoB100 apolipoprotein chemically *in vivo* modified with MAA adducts. Usually, more than 75% of the free lysine residues of apoB100 have been modified with MAA adducts	In‐house	[25, 50, 51, 52]
MAA‐BSA	Bovine serum albumin modified with malondialdehyde‐acetaldehyde. Usually more than 75% of the free lysine residues of BSA have been modified with MAA adducts	In‐house	[6, 25, 44, 45, 46, 47]
PCho	Phosphocholine modified Keyhole limpet hemocyanin (KLH). PCho is a cross‐reactive virulence factor of several bacteria, *for example*, *Streptococcus pneumoniae, Haemophilus influenzae, Pseudomonas aeruginosa and Neisseria species*. KLH is used as a carrier molecule	Biosearch Tech. Novato, CA, USA Cat. no PC‐1013‐5	[53, 54, 55]
Rpg44	Gingipain A hemagglutinin domain Rgp44. Cross‐reactive virulence factor of gram‐negative periodontal disease bacteria *Porphyromonas gingivalis*	In‐house	[26]

### Statistics

All statistical analyses were carried out using IBM SPSS Statistics software 25.0 (IBM, Armonk, NY, USA). Wilcoxon signed rank test was used for statistical analyses. Pearson's correlation test was used for correlations. p‐values <0.05 were considered as statistically significant.

## RESULTS

### Clinical characteristics

The clinical characteristics of the subjects and the effect of RYGB surgery on the clinical variables have been described earlier in detail [22]. In general, body mass index (BMI) and diastolic blood pressure decreased significantly after the surgery. Also, fasting plasma glucose, glycosylated hemoglobin (HbA1C), plasma total triglycerides and plasma total LDL cholesterol levels decreased significantly after the RYGB surgery.

### Fecal protein and immunoglobulin levels before and after RYGB surgery

**Fig. 1:**
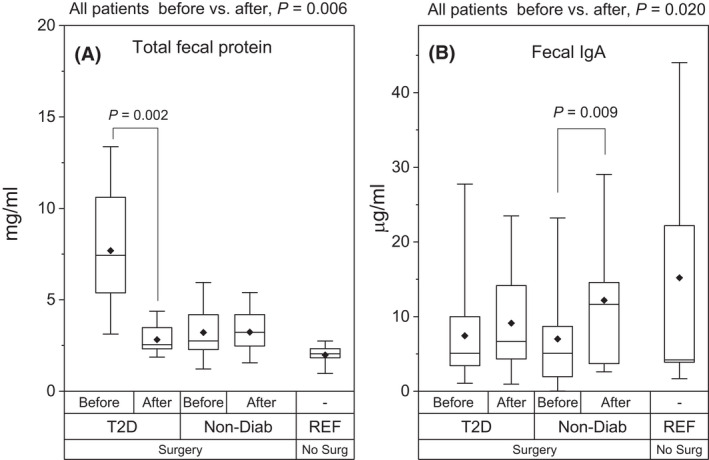
Total fecal protein and fecal IgA levels. Total fecal protein (panel A) and immunoglobulin A (IgA; panel B) in obese individuals with type 2 diabetes (T2D, *N* = 16) and non‐diabetic individuals (Non‐Diab, *N* = 14) before and after Roux‐en‐Y (RYGB) surgery. Reference subjects were non‐obese, non‐diabetic, individuals who were not operated (REF, *N* = 6). The box plots represent 25%, 50% and 75%, and the whiskers represent 1% and 99% distribution of the original values. The solid diamonds represent the mean values.

The total fecal protein levels and total fecal IgA levels are presented in Fig. 1 – the reference group is presented for comparison. T2D group had significantly higher total fecal protein levels before the surgery compared to ND study subjects and reference subjects (Fig. 1A). After the surgery, the total fecal protein levels were similar between the study groups. When analyzing all patients (T2D and ND) together, the total fecal protein concentration decreased significantly after surgery (p = 0.006).

The total fecal IgA concentrations increased significantly in ND individuals after surgery (Fig. 1B). The total level of IgA was significantly elevated after surgery also when analyzing the whole study population (T2D and ND) together (p = 0.020). No significant changes were discovered in total fecal IgG or IgM immunoglobulin concentrations before and after the surgery between study groups, and the levels were comparable to those of the reference individuals (data not shown).

### Fecal levels of antibodies to oxLDL before and after RYGB surgery

**Fig. 2:**
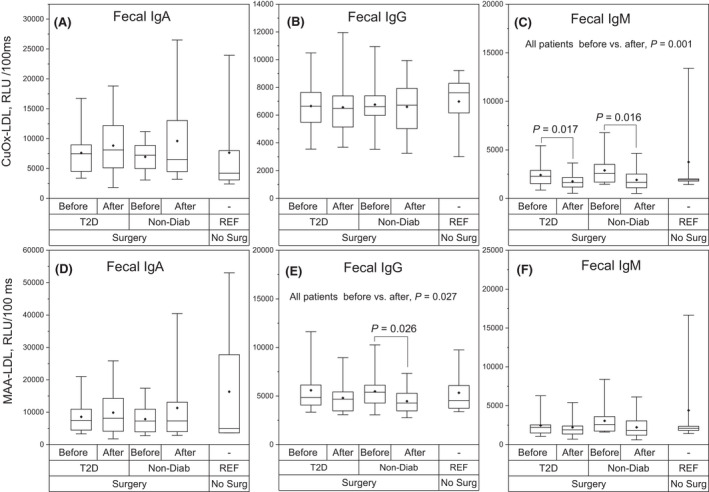
Fecal antibody levels to CuOx‐LDL and MAA‐LDL. Specific fecal IgA, IgG and IgM antibody levels to oxLDL in obese individuals with type 2 diabetes (T2D, *N* = 16) and in obese non‐diabetic individuals (Non‐Diab, *N* = 14) before and after Roux‐en‐Y (RYGB) surgery. Reference subjects were non‐obese, non‐diabetic, individuals who were not operated (REF, *N* = 6). Specific fecal antibodies to copper‐oxidized low‐density lipoprotein (CuOx‐LDL) are shown in upper panels A, B and C. Specific fecal antibodies to malondialdehyde‐acetaldehyde‐modified low‐density lipoprotein (MAA‐LDL) are shown in lower panels D, E and F. The box plots represent 25%, 50% and 75%, and the whiskers represent 1% and 99% distribution of the original values. The solid diamonds represent the mean values. RLU, relative light unit.

Fecal antibody levels to CuOx‐LDL and MAA‐LDL are presented in Fig. 2. No statistically significant changes were observed in specific IgA antibody levels to oxLDL epitopes after the RYGB surgery (Fig. 2A,D). There were also no significant changes in IgG antibody levels to CuOx‐LDL (Fig. 2B). However, specific IgG antibody levels to MAA‐LDL decreased significantly after the RYGB surgery in ND individuals (Fig. 2E). Specific IgM antibody levels to CuOx‐LDL decreased significantly after the RYGB surgery in both T2D and ND study groups (Fig. 2C). IgM levels to CuOx‐LDL decreased significantly also when analyzing the whole study population (p = 0.001). Fecal IgM antibody levels to MAA‐LDL were not affected by RYGB surgery (Fig. 2F), but fecal IgM antibody levels to MAA‐BSA decreased significantly when analyzing the whole study population (p = 0.024, data not shown).

### Fecal levels of antibodies to bacterial virulence factor Rgp44 and PCho before and after RYBG surgery

**Fig. 3:**
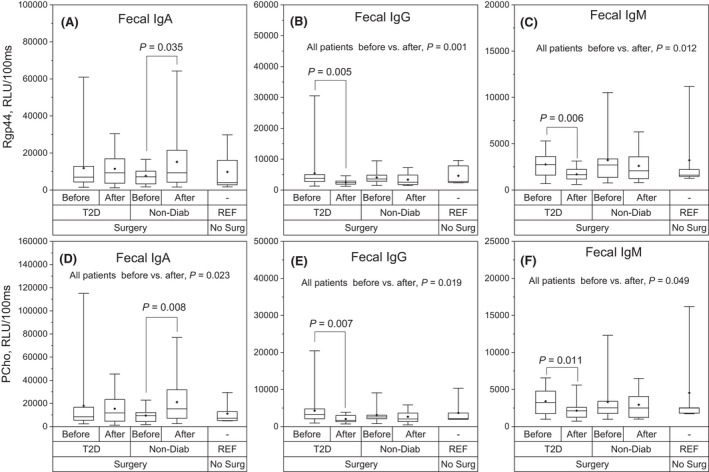
Specific fecal antibody binding to bacterial virulence factor Rgp44 and PCho. Specific fecal IgA, IgG and IgM antibody levels to cross‐reactive virulence factors in obese individuals with type 2 diabetes (T2D, *N* = 16) and in obese non‐diabetic individuals (Non‐Diab, *N* = 14) before and after RYBG surgery. Reference subjects were non‐obese, non‐diabetic, individuals who were not operated (REF, *N* = 6). Specific fecal antibodies to *Porphyromonas gingivalis* gingipain A hemagglutinin domain Rgp44 are shown in upper panels A, B and C. Specific fecal antibodies to phosphocholine (PCho) are shown in lower panels D, E and F. The box plots represent 25%, 50% and 75%, and the whiskers represent 1% and 99% distribution of the original values. The solid diamonds represent the mean values. RLU = relative light unit.

Specific fecal antibody binding to bacterial virulence factor Rgp44 and PCho is presented in Fig. 3. IgA to Rgp44 and PCho increased significantly after RYGB surgery in ND individuals (Fig. 3A,D). Also, when analyzing the whole study population, IgA to PCho increased significantly (p = 0.023). Both IgG and IgM to Rgp44 and PCho decreased significantly in T2D individuals and the whole study population after the surgery (Fig. 3B,C,E,F).

### Connection between fasting plasma glucose levels and fecal immunoglobulin levels before RYGB surgery

We also wanted to investigate if fecal immunoglobulin levels were linked with impaired glucose metabolism. Figure S1 shows that before RYGB surgery, ND individuals had significant negative associations with fecal immunoglobulins.

### Connection between body weight and plasma lipids with fecal immunoglobulin levels after RYGB surgery

To further investigate if fecal immunoglobulin levels were linked to obesity, body weight and BMI of the patients were investigated together with fecal antibody levels. Data are presented in Fig. S2. Lowest concentration of fecal IgA was correlated with highest body weight in T2D patients (Fig. S2A). Also, fecal IgM to MAA‐BSA and CuOx‐LDL were significantly negatively associated with body weight and BMI in the whole study population (Fig. S2B–E).

Finally, we wanted to investigate if fecal immunoglobulin levels were associated with plasma lipoprotein metabolism, and we investigated plasma lipids together with fecal immunoglobulin levels. Data are presented in Figs S3–S6. Fecal IgG and IgM to specific epitopes were negatively associated with total plasma cholesterol and LDL cholesterol after RYGB surgery (Figs S3 and S4). Fecal IgM and IgG to specific epitopes were negatively associated with high‐density lipoprotein (HDL) cholesterol in T2D patients before surgery, but no association was seen after the surgery.

## DISCUSSION

RYGB surgery is an effective surgical procedure for sustained weight loss in individuals with obesity and comorbidities. In addition to multiple beneficial outcomes, it has also been associated with adverse health effects, such as bone loss [27], malabsorption [28], hypoglycemia [29], formation of gallstones [30] and kidney stones [31] and development of systemic autoimmune diseases [32]. The impact of RYGB surgery on the gut mucosal immune system is largely unknown. In the present study, we showed that 6 months after the RYBG surgery, levels of secreted gut total IgA antibodies were elevated, while IgM natural antibodies were significantly decreased. There are only few studies on secretory fecal antibodies published in humans, and a majority of them are focused on secretory IgA. No prior study has reported the effect of bariatric surgery on fecal total or specific immunoglobulins. Natural antibodies of mucosal sites are considered to have an important role in first‐line defense and maintenance of normal homeostasis and highlight the importance of present findings.

RYGB surgery has various effects on the cells of the immune system, including those involved in antibody production. After surgery, follicular helper T cells have been shown to promote development of regulatory B cells by secretion of IL‐10 and TGF‐β [33], and this is likely to contribute to the lower systemic inflammatory status seen in these individuals after the surgery. Follicular helper T cell levels were also associated with better response (reduced clinical symptoms of obesity and diabetes) to RYGB surgery [33]. However, it has been reported that individuals after RYGB surgery have a decreased number of IL‐6‐producing B cells [34]. IL‐6 is a cytokine with multiple phenotypic expressions and numerous roles in both innate and adaptive immune systems, and in metabolic homeostasis [35]. Antibodies are produced by B cells, and IL‐6 is needed for their proliferation and T‐cell‐dependent isotype switching; thus, a decrease in IL‐6 can result in decreased antibody production and maturation after RYGB surgery. In this study, we showed that fecal IgM and IgG antibody levels to oxidized epitopes in oxLDL, MAA‐LDL and MAA‐BSA were decreased after RYGB surgery. The IgM antibodies binding to oxLDL, MAA‐LDL and MAA‐BSA in the present study are phenotypically likely to be natural antibodies, since oxLDL has been well documented to attract polyreactive IgM natural antibody binding by its negatively charged oxidized lipid antigenic moieties [3, 36, 37]. We have earlier cloned human antibodies binding to MAA adducts from fetal cord blood lymphocytes and showed that they have no heavy chain (VH) and light chain (VL) mutations and are germline in origin characteristic for natural antibodies [5]. It can be speculated that the natural IgM levels are elevated as a compensatory mechanism to help balance gut homeostasis in individuals with obesity, and the surgery normalizes the condition followed by a decrease in natural IgM levels after the surgery. However, this hypothesis is not supported by the post‐operative associations of low IgM levels with high body weight and BMI seen in our study subjects, and the decrease in gut natural antibodies after RYGB surgery may thus be a negative outcome of the surgery.

There are only a few reports on autoantibodies in blood samples in individuals undergoing RYGB surgery. A decrease in rheumatoid factor (RF) antibody levels and anti‐cardiolipin antibody levels has been reported [38], and it should be noted that a majority of RF antibodies are IgM isotype. Also, individuals developing anti‐nuclear antibodies with homogeneous and centriole patterns after the surgery have been reported [38]. A report of four patients developing systemic lupus erythematosus, antiphospholipid syndrome and rheumatoid arthritis within a year after the RYGB surgery [32] and a case report of a patient developing histiocytic necrotizing lymphadenitis after two years of surgery have been published, and these reports further emphasize the role of gut immune system in autoimmune diseases. The reduction of natural antibodies seen in the present study can be speculated to increase the risk of developing autoimmune diseases.

Secretory IgA is the most abundant immunoglobulin in the gut and other mucosal membranes. It is synthesized by plasma cells in the lamina propria and exported through the epithelium into the gut secretions. Many cytokines, including IL‐4, TGF‐β, IL‐5, IL‐6, IL‐10, are needed for the secretory IgA production [39]. Our data showed that total fecal IgA levels increased markedly after RYGB surgery in the whole study population but was more pronounced in the ND study group. Increased levels of secretory IgA have been documented in individuals with active ulcerative colitis and Crohn's disease, and the amount of fecal IgA was positively associated with the inflammatory bowel disease activity [40]. In our study, the total fecal IgA levels and specific IgA to Rgp44 and PCho were increased. Natural polyreactive secretory IgA antibodies in human saliva and colostrum samples have been described earlier [41], and it is known that commensal bacteria are normally coated by IgA in human feces, suggesting that secretory IgA is an important factor involved in maintaining the normal mucosal homeostasis. The data of the present study show that natural polyreactive secretory IgA antibody levels, *that is* IgA to oxidized epitopes, remain unchanged after the RYGB surgery and that the increase in the total secretory IgA may have resulted from adaptive immune system rather than from the natural antibodies of the normal mucosal innate immune system. Secretory IgA may restrict the inflammatory response by not activating the complement system. In our prior study, RYGB surgery resulted in the increase of several fecal markers of inflammation including calprotectin and lipopolysaccharide [22]. Thus, the increase of total fecal IgA may be a compensatory anti‐inflammatory mechanism to resist the inflammation caused by RYGB surgery. It is of interest that in ND individuals before the surgery, total fecal IgA level was negatively correlated with fasting plasma glucose, while after the surgery, total fecal IgA level was negatively correlated with the body weight. This could suggest that the gut IgA has links to energy metabolism.

Reduction in plasma glucose and lipid levels is an important advantage of RYGB surgery and was observed in both T2D and ND study groups in the present study and contributes to a better cardiovascular risk profile after RYGB surgery. We observed that after RYGB surgery, low fecal natural antibody levels were associated with high plasma total cholesterol and LDL cholesterol levels. Similar association with HDL cholesterol levels was observed only before the surgery and not after the surgery. Impaired digestion and malabsorption are known to occur due to reduction of volume of the food intake and surgical elimination of a part of the digestive tract. According to our data, high fecal antibody levels to oxLDL seem to be associated with low plasma lipid levels. It can be speculated that high antibody levels to oxidized lipids may interfere with lipid absorption in the gut, resulting in a decrease in plasma lipid levels. However, there are no data or previous publications to support this hypothesis.

As we demonstrated in our previous paper [22], fecal protein concentrations were over two‐fold higher in the T2D group before the surgery, compared with both the ND group and the non‐surgery individuals. We show and discuss these results also here as we can now rule out elevated immunoglobulin levels as the cause of the high fecal total protein concentration in T2D individuals with obesity. The nature of elevated proteins is currently unknown and requires further study.

Antibodies against oxLDL and oxidation‐specific epitopes [42, 43] have been linked with several diseases including atherosclerotic coronary artery disease, obesity, diabetes and autoimmune diseases [8, 9, 44, 45]. In recent years, we have investigated antibodies binding to cross‐reactive epitopes shared by oxLDL and bacterial virulence factors [7, 9, 46, 47]. In the present study, before RYGB surgery low levels of fecal IgA, IgM and IgG antibodies to Rgp44 were clearly associated with high levels of fasting plasma glucose levels in ND individuals, suggesting a link to glucose metabolism. We have earlier observed similar associations between fasting blood glucose levels and plasma IgA to PCho in a population‐based cohort representative of middle‐aged Finns [48]. We have also reported that saliva levels of IgA to Rgp44 are increased in individuals with acute coronary disease [9], and plasma IgA levels to PCho are associated with long‐term cardiovascular disease risk [8]. Here we found that fecal IgM to oxLDL, Rpg44 and PCho was decreased after the RYGB surgery. A recent systematic review on circulating antibodies to oxLDL and coronary artery disease (CAD) found that IgM antibodies to oxLDL indicated protection from more severe CAD and for IgG antibodies studies reported conflicting results [49]. If IgM antibodies to oxLDL possess cardioprotective properties, the results of current study may indicate undesirable effects of RYGB surgery. The marked reduction in plasma lipid and glucose levels as well as reduced blood pressure, however, are likely to overcome the putative negative cardiovascular effects associated with decreased IgM antibody levels to oxLDL seen in the present study.

This study has its limitations, which are listed in detail in our other paper [22]. The size of the study cohort was smaller than expected, and an ideal control group is lacking. Also, it would have been beneficial to collect follow‐up samples after weight loss had been stabilized.

## CONCLUSION

In conclusion, RYGB surgery influences gut immune system, resulting in a decrease in fecal natural IgM to oxLDL and an increase in fecal total IgA levels. After surgery, lower levels of natural IgM to oxLDL were associated with higher body weight, BMI and lipid levels in ND individuals. Natural antibodies are known to provide protection against bacterial, viral and fungal infections, and these findings suggest that RYGB surgery may modify the natural antibody levels in a non‐desirable direction. Further studies should be designed to investigate if the putative positive effects on increased total fecal IgA levels can offset the putative negative effects of lower natural IgM antibodies.

###  

N.I. wrote the first draft of the manuscript, performed statistical analyses and part of the experimental work. J.H., M.J.S., V.K., S.H. contributed to the conception of the idea, design of the study and critically evaluated the manuscript. R.A., AE.N., M‐A.H. and K.A. were involved with the experimental work and critically evaluated the manuscript. M.L. and P‐H. G. participated in the interpretation of the results and critically evaluated the manuscript. All authors approved the final content of the manuscript. Yrjö Jahnsson Foundation (20197174), Academy of Finland (275614, 316664, and 315568), Novo Nordisk Foundation (#NNF OC0013659), Signe and Ane Gyllenberg Foundation, Folkhälsan Research Foundation, Helsinki University Central Hospital Research Funds, the Diabetes Research Foundation, the Päivikki and Sakari Sohlberg Foundation, the Finnish Foundation for Cardiovascular Research, the Northern Finland Health Care Support Foundation, the Finnish Medical Foundation, and Wilhelm and Else Stockmann Foundation supported this study. We thank study nurses and coordinators for their assistance in conducting the study visits.

## CONFLICT OF INTEREST

P‐H.G. has received research grants from Eli Lilly and Roche, lecture fees from Astellas, Astra Zeneca, Boehringer‐Ingelheim, Eli Lilly, Elo Water, Genzyme, Medscape, MSD, Mundipharma, Novartis, Novo Nordisk, PeerVoice, Sanofi and SCIARC. He is an advisory board member for AbbVie, Astellas, Boehringer‐Ingelheim, Eli Lilly, Janssen, Medscape, MSD, Mundipharma, Novartis, Novo Nordisk, and Sanofi. All other authors declared that they had no competing interest associated with this manuscript.

## Supporting information


**Figure S1.** Association between fecal immunoglobulin levels and fasting plasma glucose levels in obese non‐diabetic individuals before the RYGB surgery.
**Figure S2.** Connection between fecal antibody levels and obesity in individuals with type 2 diabetes (T2D, *N* = 16) and non‐diabetic individuals (Non‐Diab, *N* = 14) after the RYGB surgery.
**Figure S3.** Associations between specific fecal IgM and IgG antibody levels with plasma total cholesterol in obese non‐diabetic individuals after the RYGB surgery.
**Figure S4.** Associations between specific fecal IgM and IgG antibody levels with LDL cholesterol in obese non‐diabetic individuals after the RYGB surgery.
**Figure S5.** Associations between specific fecal IgM antibody levels with HDL cholesterol in obese individuals with type 2 diabetes before and after the RYGB surgery.
**Figure S6.** Associations between specific fecal IgG antibody levels with HDL cholesterol in obese individuals with type 2 diabetes before and after the RYGB surgery.
