# Supplementary material for: Total fecal IgA levels increase and natural IgM antibodies decrease after gastric bypass surgery
Source: APMIS. 2022 Aug 26;130(11):637–46. doi: 10.1111/apm.13268 (PMC9805076; doi:10.1111/apm.13268)
Supplement: Supplementary file 1 — Figure S1. Association between fecal immunoglobulin levels and fasting plasma glucose levels in obese non‐diabetic individuals before the RYGB surgery. Figure S2. Connection between fecal antibody levels and obesity in individuals with type 2 diabetes (T2D, N = 16) and non‐diabetic individuals (Non‐Diab, N = 14) after the RYGB surgery. Figure S3. Associations between specific fecal IgM and IgG antibody levels with plasma total cholesterol in obese non‐diabetic individuals after the RYGB surgery. Figure S4. Associations between specific fecal IgM and IgG antibody levels with LDL cholesterol in obese non‐diabetic individuals after the RYGB surgery. Figure S5. Associations between specific fecal IgM antibody levels with HDL cholesterol in obese individuals with type 2 diabetes before and after the RYGB surgery. Figure S6. Associations between specific fecal IgG antibody levels with HDL cholesterol in obese individuals with type 2 diabetes before and after the RYGB surgery. [file APM-130-637-s001.docx]

**Supplemental Figure 1.**

**Supplemental Figure 1.** Association between fecal immunoglobulin levels and fasting plasma glucose levels in obese non-diabetic individuals before the RYGB surgery. Panels A, B and C: fecal IgA, IgM and IgG antibody levels to cross reactive antigen *Porphyromonas gingivalis* gingipain A hemagglutinin domain Rgp44. Panels C, E and F: Total fecal IgA, IgM and IgG immunoglobulin levels versus fasting plasma glucose.

**Supplemental Figure 2**

**Supplemental Figure 2.** Connection between fecal antibody levels and obesity in individuals with type 2 diabetes (T2D, N=16) and non-diabetic individuals (Non-Diab, N=14) after the RYGB surgery. Correlation of tertiles of total fecal IgA levels (tertile 1 is the lowest and tertile 3 the highest IgA concentration) with body weight (panel A). Fecal IgM binding to malondialdehyde-acetaldehyde-modified bovine serum albumin (MAA-BSA) versus body weight (panel B), and versus body mass index, BMI (panel D). Fecal IgM binding to copper-oxidized low-density lipoprotein (CuOx-LDL) versus body weight (panel C), and versus body mass index, BMI (panel E).

**Supplemental Figure 3.** Associations between specific fecal IgM and IgG antibody levels with plasma total cholesterol in obese non-diabetic individuals after the RYGB surgery.

**Supplemental Figure 4.** Associations between specific fecal IgM and IgG antibody levels with LDL cholesterol in obese non-diabetic individuals after the RYGB surgery.

**Supplemental Figure 5.** Associations between specific fecal IgM antibody levels with HDL cholesterol in obese individuals with type 2 diabetes before and after the RYGB surgery.

**Supplemental Figure 6.** Associations between specific fecal IgG antibody levels with HDL cholesterol in obese individuals with type 2 diabetes before and after the RYGB surgery.
